# Long-term follow-up after viral myocarditis established by endomyocardical biopsy: Predictors of mortality

**DOI:** 10.1186/1532-429X-13-S1-M7

**Published:** 2011-02-02

**Authors:** Stefan Grün, Tim Schäufele, Tülay Derin, Eva-Maria Kispert, Karin Klingel, Reinhard Kandolf, Udo Sechtem, Heiko Mahrholdt

**Affiliations:** 1 Division of Cardiology, Robert-Bosch-Medical Center, Stuttgart, Germany; 2 Department of Molecular Pathology, University of Tübingen, Tübingen, Germany

## Introduction

Myocarditis is a frequent cardiac disease occurring in all ages that is difficult to diagnose. However, new procedures such as cardiac magnetic resonance imaging (CMR) and biventricular endomyocardial biopsy with histological, immunohistochemical and microbiological work-up have revolutionized the diagnosis of myocarditis in the last years. Nevertheless, the long-term mortality after viral myocarditis established by endomyocardical biopsy, as well as the predictors of long-term mortality are still not known.

## Methods

Long-term clinical follow-up (more than 5 years) of 80 patients with viral myocarditis established by endomyocardical biopsy and initial contrast-CMR.

## Results

A median follow-up period of 6.5 years and a follow-up rate of 91% (n = 73) could be achieved in the study. The mean age of the studied patients was 52 years (male n= 51). 16 of the 73 patients died during the follow-up. This represents a mortality of 22% within 6.5 years (about 3.5% per year). A cardiac cause of death was found in 11 patients. The remaining 5 patients died from non-cardiac causes.

Univariate analysis revealed the presence of late gadolinium enhancement (LGE) as the best predictor for death in the follow-up period (p = 0.014), followed by LV enlargement in the initial CMR (p = 0.03).

In contrast, neither the initial LV function nor the presence of clinical symptoms (angina p = 1.0 vs. heart failure, p = 0.4 vs. other p = 0.7) or the type of virus was significant (p= 0,3) in the current analysis.

## Conclusions

In the follow up period of 6,5 years the overall mortality after biopsy proven viral myocarditis is more than 20%. The best predictors of death were the presence of LGE and a dilated LV at the initial presentation.

